# Epidemiology and risk factors of voluntary pesticide poisoning in Morocco (2008-2014)

**DOI:** 10.4178/epih.e2017040

**Published:** 2017-09-01

**Authors:** Zineb Nabih, Latifa Amiar, Zakaria Abidli, Maria Windy, Abdelmajid Soulaymani, Abdelrhani Mokhtari, Rachida Soulaymani-Bencheikh

**Affiliations:** 1 Laboratory of Genetics and Biometry, Faculty of Science, Ibn Tofail University, Kenitra, Morocco; 2 Faculty of Science and Technology, University Abdelmalek Essaadi, Tangier, Morocco; 3 Moroccan Anti-Poison and Pharmacovigilance Center, Rabat, Morocco; 4 Mohammed V University, Rabat, Morocco

**Keywords:** Poisoning, Voluntary, Epidemiology, Pesticide, Risk factors, Morocco

## Abstract

**OBJECTIVES:**

To determine the epidemiological profile and risk factors of voluntary poisoning by pesticides.

**METHODS:**

A retrospective analysis was conducted of all cases of voluntary poisoning by pesticides registered at the AntiPoison and Pharmacovigilance Center of Morocco between January 2008 and December 2014.

**RESULTS:**

During the study period, 2,690 cases of acute pesticide poisoning were registered. The region of Rabat-Salé-Zemmour-Zaer accounted for the largest proportion, with 598 cases. The average age of the patients was 24.63±10.29 years. The sex ratio (female-to-male) was 0.45. Adults and teenagers were most affected by this type of poisoning, with 1,667 cases (62.0%) and 806 cases (30.0%), respectively. Suicide attempts accounted for 98.4% of the cases (2,469 cases). Pesticide poisoning occurred more often in urban zones (64.8%). Insecticides were incriminated in 14.0% of cases, with a mortality rate of 4.2%. Among the 1,635 patients for whom the outcomes were known, 154 died, corresponding to a mortality rate of 5.7%.

**CONCLUSIONS:**

Voluntary intoxication by pesticides presents a real scourge that affects public health, and in this study, we developed an epidemiological profile of this phenomenon. Nevertheless, this study has limitations in that it did not evaluate the impact of the socioeconomic and psychological factors that are important contributors to this type of poisoning.

## INTRODUCTION

Pesticides constitute a very heterogeneous group of chemical substances designed to destroy unwanted plants, insects, and rodents, and they mainly include weed-killers, fungicides, insecticides, acaricides, nematicides, and rodenticides. These phytosanitary products all possess a certain level of toxicity for humans, with variable intensity [1].

In parts of some developing countries, pesticide poisoning causes more deaths than infectious diseases. The use of pesticides is poorly regulated and often dangerous; moreover, they are easily available, which makes them a popular method of self-harm [2]. According to a report of the World Health Organization, the number of annual pesticide poisoning cases is estimated to be between 1 and 5 million, including several thousand fatal cases [3].

The proposed link between human exposure to pesticide and nervousness, anxiety, and depression, leading farmers to suicidal acts, is an important health concern in rural populations in Brazil [4]. A total of 11 studies on depression and 14 studies on suicide showed links to the use of pesticides. Increased risks of depression or other psychiatric disorders have been associated with previous pesticide poisoning in 5 studies, with statistically significant odds ratios (ORs) ranging from 2.08 to 5.95 [5]. In Morocco, the voluntary use of pesticides for suicide or criminal purposes has become increasingly alarming. According to a study conducted between 1989 and 2007 at the Moroccan Anti-Poison and Pharmacovigilance Center (MAPPC) of 10,332 collected cases of acute pesticide poisoning, 55.15% voluntary poisoning, and 99.00% of those cases were suicide attempts [6].

The aim of this study was to describe the demographic and clinical characteristics and outcomes of voluntary poisoning by pesticides reported to the MAPPC between 2008 and 2014, as well as to analyze the risk factors associated with voluntary pesticide poisoning.

## MATERIALS AND METHODS

The MAPPC is a public institution established by the Ministry of Health with the mandate of managing poisonings in Morocco. It is dedicated to its functions of vigilance and announcing sanitary alerts. It collects information on poisonings and creates national databases. This study was conducted using data collected by the MAPPC.

The present study is a retrospective analysis of all cases of voluntary poisoning by pesticides collected at the toxicological information unit of the MAPPC over a period of 7 years, from January 2008 to December 2014. Concerning the data processing, we used some descriptive statistical tools: namely, frequencies, averages, specific fatality rate, and mortality rate.

A descriptive analysis was conducted of the characteristics of the intoxicated patients (sex, age, and place of residence), characteristics of the toxicant (product family), and characteristics of the poisoning (type of poisoning, circumstance, symptomatology, gradation, and outcomes). Age was analyzed according to the distribution of the International Programme on Chemical Safety [7]: child, 5-14 years; adolescent, 15-19 years; adult, 20-74 years; elderly, 75 years of age or older.

Severity was evaluated using the poisoning score severity scale [8]: grade 0 (none), absence of sign or symptom; grade 1 (minor), symptoms slight, transient, and disappearing spontaneously; grade 2 (moderate), prolonged symptoms; grade 3 (severe), serious or life-threatening symptoms; grade 4 (fatal), death.

The contingency test (chi-square) and the calculation of ORs allowed us to study the associations between the studied variables and outcomes. The fatality and mortality rates were also calculated in order to characterize the gravity of the problem in a precise manner.

## RESULTS

**Figure 1.**
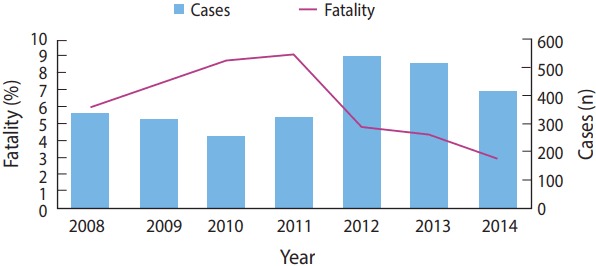
Distribution of voluntary poisoning cases (n=2,690) by pesticides according to years and specific lethality (p<0.001).

This study analyzed 2,690 cases of voluntary poisoning by pesticides reported to the MAPPC during the period between 2008 and 2014. The distribution of these cases varied slightly across this time interval, with a peak of 541 cases observed in 2012. However, the peak of the specific fatality rate occurred in 2011, with a percentage of 9.09% (Figure 1).

**Figure 2.**
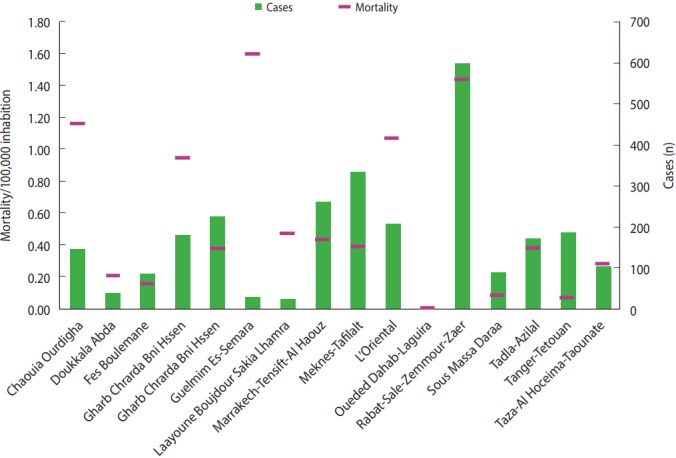
Distribution of voluntary poisoning cases (n=2,690) by pesticides according to region and mortality (p<0.001).

The highest rate of voluntary poisoning by pesticides was recorded in the Rabat-Sale-Zemmour-Zaer region with 598 cases, followed by the Meknes-Tafilalt region with 332 cases; however, the highest mortality was recorded in the Guelmim Es-Smara region with 1.60 per 100,000 inhabitants, followed by Rabat-Sale-Zemmour-Zaer with 1.43 per 100,000 inhabitants. The least affected region was Oueded Dahab-Lagouira, with only 3 cases (Figure 2).

Table 1 presents the epidemiological characteristics of the studied population and the characteristics of the poisonings. Voluntary poisoning by pesticides mainly took place in adults (62.0% of cases), with a specific fatality of 6.5%, followed by teenagers (30.0%), but the highest fatality rate was observed in toddlers (25.0%). The average age of the intoxicated patients was 24.63± 10.29 years. Females were most often affected 66.5% (p< 0.001); however, a higher fatality rate was observed in males (7.8%), and the sex ratio (male/ female) was 0.45. Intoxication cases of urban origin accounted for 64.8% of all cases, with a specific fatality of 5.9%. Symptomatic patients accounted for 73.5 % (p< 0.001), and the toxicant was ingested orally in 97.8% of cases. The severity of voluntary poisoning was predominantly moderate (grade 2) (44.1%). Among the 1,635 patients for whom the outcomes were known, 154 died, corresponding to a fatality rate of 5.7%. Insecticides were incriminated in 14.0% of cases, with a fatality rate of 4.2%.

The distribution of the poisoning cases according to circumstances showed that suicide attempts accounted for 98.4% of cases (2,469 cases), and adult females were the most commonly affected group, accounting for 39.5% of cases (992 cases), followed by teenager girls (24.5%, 616 cases) and female children (4.5%, 114 cases). Criminal circumstances, addiction, and abortion accounted for 0.6, 0.5, and 0.5% of cases, respectively (Table 2). Insecticides were most commonly used in suicide attempts, in 46.4% of cases, followed by rodenticides (37.1% of cases) (Table 3).

**Figure 3.**
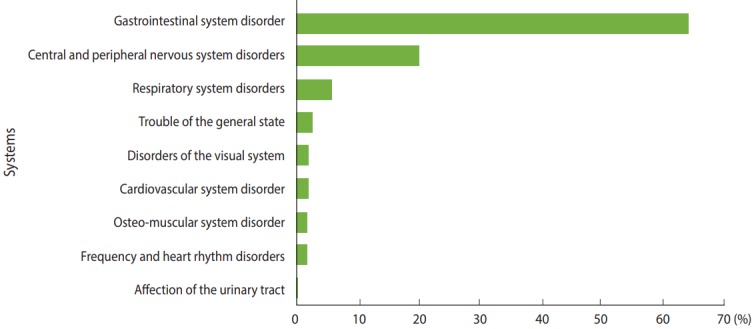
Distribution of clinical manifestations among patients (n=596) intoxicated voluntary by pesticides according to the system or organ concerned (p<0.001).

Figure 3 is a synthesis of the clinical manifestations by the category of effects according to the system or the affected organ. The number of signs observed was 591, distributed throughout different systems. Clinical signs specific to the gastrointestinal system and the central and peripheral nervous system were found with a frequency of 65.9% (n= 383) and 20.7% (n= 120), respectively.

In order to highlight the factors influencing the prognosis of patients voluntarily poisoned by pesticides, we studied the effects of sex, age, place of residence, clinical status, and type of poisoning. Sex and clinical status were risk factors with a significant influence on the health status of the studied patients. Females had twice as high a risk of death as males (relative risk [RR], 2.10; 95% confidence interval [CI], 1.23 to 2.43). Furthermore, asymptomatic patients had almost twice as high of a risk of progressing to death as those with clinical signs (RR, 1.74; 95% CI, 1.16 to 2.62) (Table 4). This analysis was performed among people whose outcomes could be confirmed.

## DISCUSSION

As in many other countries, in Morocco, voluntary poisoning by pesticides is responsible for many deaths each year, and is considered to be a major public health issue.

In our study, we recorded 2,690 cases of voluntary poisoning by pesticides between 2008 and 2014. Suicide attempts accounted for 98.4% of these poisonings, while poisonings with criminal intent, with the goal of causing abortion, and due to drug addiction represented only a small percentage. This rate is very high compared to what has been reported in the literature [9,10], but it is also underestimated in relation to the reality of the phenomenon. Studies on suicide attempts and suicides have shown that every year, nearly 1 million people die of suicide around the world [11]. Suicide attempts are a relatively common cause of hospitalization in emergency departments [12].

In Morocco, this practice is a taboo subject, as suicide attempts are forbidden in the religious and social context of Moroccan society; once perpetrated, the family of the subject lives in unspoken shame.

In our series of cases, of the 2,690 cases of voluntary poisoning by pesticides, 2,469 were suicide attempts. A study found an association between pesticide exposure and suicide in Brazil [13]. International studies, most notably in the US [14], France [15], Costa Rica [16], and South Africa [17], have shown a greater risk of psychiatric problems, mainly depression, in people exposed to pesticides, especially those who have suffered from pesticide poisoning.

In our study, voluntary poisoning affected both sexes, with a female predominance and a higher fatality in males. Our results agree with those of numerous studies, both domestic and international [18-20], which have reported that the rates of suicide attempts were 2-3 times higher among girls than among boys. The average age of the patients was 24.63 ± 10.29 years. Adults and teenagers were most commonly affected. According to Amos et al. [21], voluntary poisoning has become an increasingly common response to emotional distress in adults; in addition to mental pathologies, certain events (such as unemployment, divorce, over-indebtedness, bereavement, and domestic violence) can trigger a loss of self-esteem, a withdrawal into oneself, and finally a depressive state that can lead to a suicide attempt. Moreover, the transition from adolescence to adulthood is accompanied by numerous physical and psychological changes. Thus, it is a delicate phase of life where teenagers are relatively vulnerable [22].

The region of Rabat-Sale-Zemmour-Zaer was the most affected by this type of poisoning; despite the very precise and strict laws regarding pesticide use in Morocco, these laws remain only a formality, since the prohibited products are still available on the national market by clandestine sales or smuggling. Therefore, we note the recommendation of certain products for a specific type of crop, or even for “domestic” use [6].

In terms of clinical parameters, important results were observed in different systems. Of note, the gastrointestinal system was affected in 65.9% of cases, and hepatotoxicity was commonly observed because the liver is a target of many toxins due to its high blood flow and its link to systemic circulation. The next most commonly affected system was the central and peripheral nervous system, with 20.7% of cases. Many studies have shown that several pesticides exert a neurotoxic action in humans and that a potential link between exposure to certain pesticides and Parkinson disease may exist, following the increase in stress markers of oxidation, in addition to other forms of neuronal degeneration and developmental abnormalities [23].

In conclusion, voluntary intoxication by pesticides presents a real scourge that affects public health, and in this study, we developed an epidemiological profile of this phenomenon. Nevertheless, this study has certain limitations, because it did not evaluate the impact of the socioeconomic and psychological factors that are important for understanding this type of poisoning. The data presented in this study are therefore suitable for use in public health endeavors with the goal of reducing fatalities. Voluntary poisoning by pesticides is preventable by strengthening awareness-raising campaigns, education, and communication about this issue in all regions of Morocco.

## Figures and Tables

**Table 1.** Characteristics of the subjects who experienced voluntary pesticide poisoning

Variables	Case (%)	Clinical status			p-value
		Recovery	Death	Unknown	
Age group					<0.001
Newborns	1 (0.1)	0	0	1	
Toddlers	4 (0.1)	2	1	1	
Children	146 (5.4)	97	6	41	
Teenagers	806 (30.0)	489	37	272	
Adults	1,667 (62.0)	1,011	108	535	
Elderly	7 (0.3)	3	1	2	
Unknown	59 (2.2)	33	1	49	
Sex					<0.001
Female	1,788 (66.5)	1,121	87	561	
Male	812(30.2)	470	63	273	
Unknown	90 (3.3)	44	4	67	
Place of residence					<0.001
Urban	1,742 (64.8)	1,124	102	510	
Rural	705 (26.2)	400	36	265	
Unknown	243 (8.7)	111	16	126	
Place					<0.001
Home	2,339 (87.0)	1,460	129	750	
Workplace	4 (0.1)	3	0	1	
Public	68 (2.5)	45	3	20	
Prison	4 (0.1)	3	0	1	
Unknown	275(10.2)	124	22	129	
Clinical status					
Asymptomatic	712(26.5)	499	31	182	
Symptomatic	1,978(73.5)	1,136	123	719	
Pathway					<0.001
Oral	2,630 (97.8)	1,595	151	858	
Inhalation	20 (0.8)	13	0	7	
Injectable	4 (0.1)	4	0	0	
Transplacental	2 (0.1)	2	0	0	
Unknown	34(1.3)	21	3	36	
Grade					<0.001
Grade 0 (none)	427(15.9)	393	0	34	
Grade 1 (minor)	254 (9.4)	237	0	17	
Grade 2 (moderate)	1,185 (44.1)	800	0	390	
Grade 3 (severe)	242 (9.0)	161	0	77	
Grade 4 (fatal)	154 (5.7)	0	154	0	
Unknown	428(15.9)	44	0	383	
Type of pesticide					<0.001
Fungicide	5 (0.2)	2	0	3	
Herbicide	18 (0.7)	12	2	4	
Insecticide	376(14.0)	201	16	159	
Rodenticide	301 (11.2)	191	12	98	
Unkown	1,990 (74.0)	1,229	124	637	

**Table 2.** Distribution of the circumstances of poisoning by age group

Circumstances	Children (n=143)		Teenagers (n=774)		Adults (n=1,586)		Elderly (n=7)		Total
	Female	Male	Female	Male	Female	Male	Female	Male	
Abortion	1	0	3	0	8	0	0	0	12
Criminal	3	2	3	0	2	6	0	0	16
Suicide attempt	114	23	616	149	992	568	1	6	2,469
Substance addiction	0	0	3	0	7	3	0	0	13
Total	118	25	625	149	1,009	577	1	6	2,510

**Table 3.** Distribution of the types of pesticides used according to circumstances

Type of pesticide	Circumstances				Total
	Abortion	Criminal	Suicide attempt	Addiction	
Fungicide	0	0	5	0	5
Herbicide	0	0	18	0	18
Insecticide	1	2	372	1	376
Pesticide	0	0	5	0	5
Rodenticide	1	2	297	0	300
Unknown	1	0	94	2	97
Total	3	4	791	3	801

**Table 4.** Effects of the studied characteristics on the outcomes of the patients

Variables		Healings	Death	RR (95% CI)	p-value
Sex	Male	470	63	2.10(1.23, 2.43)	10.02
	Female	1,121	87	1.00 (reference)	
Age (yr)	>15	1,504	146	0.73 (0.33,1.61)	0.59
	≤15	98	7	1.00 (reference)	
Origin	Urban	1,124	87	0.99 (0.67,1.47)	0.002
	Rural	400	63	1.00 (reference)	
Clinical status	Symptomatic	1,136	123	1.74 (1.16, 2.62)	7.29
	Asymptomatic	499	31	1.00 (reference)	
Type of poisoning	Isolated	1,613	154	0.47 (0.64, 3.58)	0.54
	Collectif	22	1	1.00 (reference)	

RR, relative risk; CI, confidence interval.
